# CD45 Isoform Expression in Microglia and Inflammatory Cells in HIV-1 Encephalitis

**DOI:** 10.1111/j.1750-3639.2006.00027.x

**Published:** 2006-10

**Authors:** Melissa A Cosenza-Nashat, Mee-Ohk Kim, Meng-Liang Zhao, Hyeon-Sook Suh, Sunhee C Lee

**Affiliations:** 1 Department of Science, BMCC, City University of New York New York, N.Y.; 2 Department of Neurology, Massachusetts General Hospital Boston, Mass.; 3 Department of Pathology, Albert Einstein College of Medicine Bronx N.Y.

## Abstract

CD45 is a membrane tyrosine phosphatase that modulates the function of the hematopoietic cells. *In vitro*, agonist antibodies to CD45RO or CD45RB isoforms have been shown to suppress microglial activation, but whether microglia *in vivo* express these isoforms in HIV encephalitis (HIVE) is unknown. Brain sections from control and HIVE were immunostained for CD45 isoforms using exon-specific antibodies (RA, RB, RC and RO). RA and RC were limited to rare lymphocytes, while RB expression was robust in microglia and inflammatory cells. RO was low in control microglia, but increased in HIVE. RO was also localized to macrophages and CD8+ T cells. Targeting CD45 *in vivo* with isoform-specific antibodies remains a therapeutic option for neuroinflammatory diseases.

## INTRODUCTION

The leukocyte common antigen (LCA: CD45) is a prototype transmembrane protein tyrosine phosphatase (PTPase) and is expressed in all nucleated hematopoietic cells (54). The CD45 protein exists as multiple isoforms as a result of alternative splicing of variable exons (4/A, 5/B and 6/C); the largest isoform (ABC) includes all three of these exons and the smallest isoform (O) lacks all three exons. Five different isoforms of CD45 (ABC, AB, BC, B and O) have been identified on human leukocytes and these can be recognized by antibodies specific to variable exons (A, B or C) or by αCD45RO (45). Although the extracellular domains differ among different isoforms, all forms share identical transmembrane and cytoplasmic domains including the phosphatase domains (52, 54).

CD45 is one of the most abundantly expressed molecules in lymphocytes (comprising approximately 10% of all surface proteins) and is crucial in lymphocyte development and antigen signaling (2, 12, 23, 54). Consequently, CD45 mutations are associated with severe combined immunodeficiency in mice and humans (5, 28, 51). In lymphocytes, CD45 is expressed in a cell subset-specific and activation-dependent manner. For instance, naïve T cells express a high molecular weight isoform (RA+/RO−) but upon activation switch to the smallest isoform (RA−/RO+) (16, 31). At the cellular level, the CD45 phosphatase targets several families of proteins, including the Src family tyrosine kinases and Janus kinases (41), resulting in positive or negative signaling (2, 4, 54). In addition to lymphocytes, recent studies demonstrate that CD45 can modulate activation and proliferation of several inflammatory cell types including granulocytes, mast cells and monocyte-lineage cells, broadening its role as a regulator of inflammatory responses (8, 20, 35, 48, 57).

In the central nervous system (CNS), microglia constitute a distinct glial cell population that is derived from hematopoietic cells in the bone marrow (17, 29, 42). As resident brain macrophages, microglia function as sentries, but when activated they can mediate tissue damage, a scenario considered for several CNS inflammatory disorders (10, 15, 27). In AIDS dementia and HIV encephalitis (HIVE), microglia and macrophages are productively infected by HIV-1 and show diffuse inflammatory activation, which ultimately leads to neuronal damage and CNS dysfunction (7, 11, 14, 43). Microglia in normal human brain express CD45 and increases in microglial CD45 expression have been detected in Alzheimer’s disease, graft-versus-host disease, multiple sclerosis, and in HIVE (1, 7, 24, 30, 33, 46). Furthermore, studies in rodent and human cells suggest that CD45 can downregulate microglial activation. For example, murine microglia devoid of CD45 expression demonstrate an over-activated phenotype (49, 50), while in human microglia, an agonist antibody (αCD45RO, clone UCHL-1) can stimulate CD45 tyrosine phosphatase activity and suppress granulocyte-macrophage colony-stimulating factor (GM-CSF) signal transduction and cell proliferation (48). CD45 also downregulates HIV-1 replication in microglia, indicating that there might be potential for targeting this phosphatase as a therapy for AIDS dementia (25).

Despite these data indicating functional importance of CD45 in microglia, the CD45 isoform expression by microglia and macrophages in HIV-1-infected human brain is not known. Furthermore, the identity of CD45 isoforms other than CD45RO on CNS-infiltrating T cells is unknown. We therefore sought to investigate changes in CD45 isoform expression in the human CNS as it pertains to HIVE and also asked whether there is cell-type or activation-dependent expression of CD45 isoforms.

## MATERIALS AND METHODS

### Patient material

Paraffin-embedded, formalin-fixed brain tissues from 22 patients were obtained from the Manhattan HIV-1 Brain Bank, National NeuroAIDS Tissue Consortium (37). Information regarding the case history and other associated systemic illnesses has been previously reported (6, 7, 58). Our patient material was distributed into three groups: HIVE (n = 9), HIV-seropositive without HIVE (HIV+, n = 6) and HIV-seronegative individuals (HIV−, n = 8). The mean ages were 45.6 ± 3.5 (HIV−), 42.5 ± 2.7 (HIV+) and 38.5 ± 2.5 (HIVE) and were not significantly different (*P* > 0.05). One HIVE and two HIV+ patients received highly active antiretroviral therapy (HAART). For HIVE, one to two regions of the frontal lobe, each demonstrating microglial nodules and/or multinucleated giant cells (MGCs), were selected for analysis. Control (non-HIVE) brain sections derived from the corresponding regions of the brain lacked focal pathology on hematoxylin and eosin. Because of the known difference between gray matter and white matter microglia (10) and the variable representation of the gray matter in each section, cell counts from white matter only were compared for analysis.

### CD45, CD3 and CD8 immunohistochemistry (IHC)

Deparaffinized slides were boiled for epitope retrieval, treated with 3% H_2_O_2_, blocked with normal goat serum, and then incubated with primary antibodies overnight at 4°C, as described (7). The antibodies used in this study, their dilutions and the methods of IHC employed are listed in Table 1. Staining with αCD45 antibodies was completed using the avidin–biotin complex method with or without the tyramide signal amplification (TSA) system (NEN Life Science Products, Boston, Mass.), as indicated. Briefly, biotin-labeled secondary methods were employed followed by exposure to horseradish peroxidase (HRP)-labeled streptavidin and then the TSA reagent as indicated by the manufacturer’s instructions. As a negative control, sections were incubated with normal mouse IgG1, 2a or 2b (BD Biosciences Pharmingen, San Diego, Calif.). IHC for CD3, CD8 and CD68 was performed as indicated in Table 1 and as described (6, 7, 58). Paraffin sections of human tonsils were used as positive controls. The specificity of the CD45 isoform antibodies was confirmed additionally by Western blot analysis (25).

**Table 1:** Antibodies and immunohistochemical methods adopted in this study.

Antigen	Antibody clone/isotype	Source	Dilution	Methods
CD45	LCA (PD7/26 + 2B11), IgG1	DAKO (Carpinteria, Calif.)	1:100	TSA*
CD45RA	HI100, IgG2b	Pharmingen (San Diego, Calif.)	1:100	TSA
CD45RB	MT4, IgG1	Pharmingen	1:100	TSA
CD45RC	MT2, IgG1	Biogenex (San Ramon, Calif.)	1:40	TSA
CD45RO	UCHL-1, IgG2a	DAKO	1:50	TSA
CD3	Polyclonal	Cell Marque Corp (Austin, Tex.)	1:250	TSA
CD8	1A5, IgG1	Vector (Burlingame, Calif.)	1:40	ABC†
CD68	KP1, IgG1	DAKO	1:600	Two-step‡
VWF	Polyclonal	DAKO	1:400	ABC

* Tyramide signal amplification. See *Materials and methods* for details.

† Avidin–biotin complex without TSA.

‡ Alkaline phosphatase-labeled secondary antibody methods without TSA.

### IHC without epitope retrieval or TSA

CD45RB and CD45RO staining on serial slides of a single paraffin block was compared using three different IHC methods with increasing sensitivity: (i) without antigen retrieval (AR); (ii) with AR; and (iii) with AR and TSA. All other experiments conducted with CD45 were performed using the most sensitive technique, that is, epitope retrieval plus TSA, unless otherwise stated.

### Double-label IHC

For double-label IHC, and occasionally for single label IHC, alkaline phosphatase-labeled anti-mouse IgG was used as the secondary, followed by 5-bromo-4-chloro-3-indolyl phosphate/nitro blue tetrazolium (BCIP/NBT) to develop color. For most double labeling, staining was performed sequentially. The first primary antibody was developed with an HRP-conjugated secondary antibody or biotin/streptavidin, and diaminobenzidine (brown), followed by the second primary antibody and then the alkaline phosphatase-labeled secondary and NBT (blue). In some instances, the TSA system was utilized for both antibodies, as outlined in Table 1. To ensure specificity and sensitivity of the staining, chromogens were switched in some double labeling studies, and more than one section of the same block was stained using the same antibody. For CD3/CD8 double-label IHC, the primary antibodies were incubated simultaneously, while the secondary antibody steps were performed sequentially.

### Quantitative analysis and statistics

Sections with single label immunostaining for CD45 isoforms (RA, RC, RB and RO) were analyzed by counting the total positive cells in the cerebral white matter of ten 400× random microscope fields per case and averaging the number. Only processes adjacent to evident nuclei were considered cells and, in HIVE cases, MGCs were counted as single cells. Cell type-specific CD45RO+ cell counts were also obtained as average numbers from six 200× fields. Cell types were distinguished based on morphology and location: parenchymal microglia (process-bearing), perivascular macrophages (elongated or round without processes) and parenchymal or perivascular lymphocytes (small and round and intensely CD45+). CD3 and CD8 cells were counted in six 400× microscope fields and expressed as average percentage (CD8+/CD3+ divided by total CD3) from four cases.

Graphs were generated using GraphPad Prism 4.0 and are presented in a box plot format where the median is indicated by a central line and the range is shown by the whiskers. The box displays the 25–75 percentiles. Statistical analyses comparing the cell counts among the three groups (HIV−, HIV+ and HIVE) were performed by one-way ANOVA followed by Bonferroni pairwise comparison. If no differences were found, *t*-*test* was performed to compare HIVE and non-HIVE (combined HIV− and HIV+) groups. *P* < 0.05 was considered significant, unless stated otherwise. All statistics were performed using GraphPad Prism.

## RESULTS

### Expression of CD45 isoforms in HIVE and controls (Figure 1)

**Figure 1:**
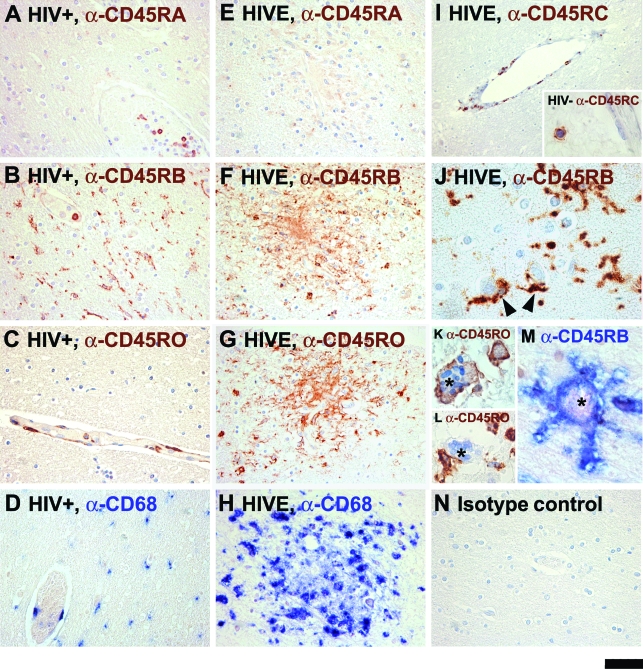
*CD45 isoform expression in HIV encephalitis (HIVE) and control brains.* Sections of the control and HIVE brains are immunostained with antibodies against CD45RA, RB, RC or RO as described in the *Materials and methods*. Results of the two non-HIVE controls groups (HIV− and HIV+) are similar (also see Figures 2 and 3E), but only HIV+ are shown here. Panels **A–D** show HIV-seropositive brains stained with αRA, αRB, αRO and αCD68. Panels **E**–**H** show serial sections of an HIVE brain depicting a microglial nodule immunostained for CD45 isoforms and CD68. The αRA- and αRC-reactive cells are mainly limited to leukocytes within the bloodstream with rare perivascular and/or parenchymal positive cells noted in both non-HIVE and HIVE brains (**A**, **E** and **I**, also see Figure 2). αRB-reactive cells are abundant in all brains and they include ramified microglial cells in HIV− (see Figure 5A) and HIV+ (**B**), as well as activated microglial cells within and outside the microglial nodules in HIVE (**F** and **J**). Multinucleated giant cells (MGCs) are also RB+ (asterisk, **M**). Panel **J** demonstrates a high power image of αRB-reactive perineuronal microglia in the gray matter (arrowheads). αRO reactivity is detected in infiltrating lymphocytes, perivascular macrophages and microglial cells in HIVE (**G**), including MGCs (asterisk, **K**). Some MGCs are RO− (asterisk: **L**). Control brains show more limited RO staining (**C** and also see Figure 3E). Microglia and macrophages in non-HIVE and HIVE brains are positive for CD68, a marker of brain macrophages (**D** and **H**). An HIV+ section treated with normal mouse IgG2a is shown as a control (**N**). Sections are counterstained with hematoxylin. The scale bar represents 50 µm in **A–H** and **N**, 100 µm in **I** and 20 µm in **I** inset and **J–M**.

Using a pan-CD45-reactive antibody we have previously shown that CD45 is expressed in normal microglia and is upregulated in HIVE (7). In this study, CD45 isoform expression was analyzed using extracellular exon-specific antibodies in control (HIV− and HIV+, HIV+ shown in A–D) and HIVE (E–N) brains with respect to several cell types (Figure 1). Results with single-label IHC are illustrated in Figure 1. αCD45RA and αCD45RC staining was limited to lymphocytes mostly within the lumina (or lumens) of the vessels. A few RA- or RC-positive lymphocytes were also present in the perivascular space and in the parenchyma of HIVE and control brains (Figure 1A,E,I). αCD45RB showed staining of ramified microglia in control brains and staining of infiltrating leukocytes and microglia including MGCs in HIVE (Figure 1B,F,J,M, also see Figure 5A for HIV−). αCD45RO staining was variable in microglia in control brains (Figure 1C, also see Figure 3F for HIV−), but was detected in lymphocytes, macrophages and microglia, including MGCs in HIVE (Figure 1G,K,L, also see Figure 3G). Control staining for macrophage/microglial (D,H) distribution and with an antibody isotype (N) is shown. The αRB reactivity in the absence of αRA or αRC reactivity indicates that the αRB-reactive isoform is B and not ABC, AB or BC (45). Therefore, of the five CD45 isoforms, those expressed in microglia, macrophages and the vast majority of lymphocytes in HIVE (and controls) are CD45RB and CD45RO.

**Figure 2:**
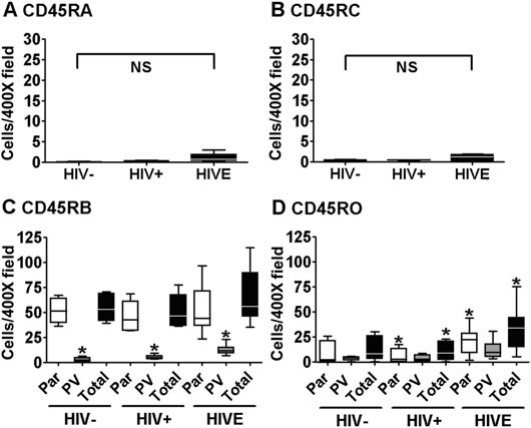
*Quantitative analysis of CD45 isoform expression in human brain.* In single-stained sections, average numbers of CD45RA, RB, RC and RO-reactive cells per 400× field were calculated and compared as described in *Materials and methods*. (**A**) CD45RA+ and (**B**) CD45RC cells were very rare relative to RB+ and RO+ cells, but showed a trend for elevation in HIVE. The numbers were not significantly different (NS) by ANOVA or *t*-*test*. (**C**) CD45RB and (**D**) CD45RO counts were obtained with respect to their location, parenchymal (Par), perivascular (PV) or total (combined Par + PV). CD45RB and RO counts are higher than RA or RC counts (see the differences in *Y*-axis scales in **A** though **D**) and CD45RB counts are higher than RO counts. The numbers in the three groups (HIVE, HIV− and HIV+) were compared in each category and showed that while perivascular CD45RB counts were significantly elevated in HIVE compared with either control group (*P* < 0.01 by ANOVA), no significant increases in parenchymal or total RB counts were seen. By contrast, RO counts were significantly elevated in HIVE (compared with HIV+ group *P < 0.05 by ANOVA) in the parenchyma. The total RO counts were also significantly elevated in HIVE compared with HIV+ (*P < 0.05 by ANOVA).

**Figure 5:**
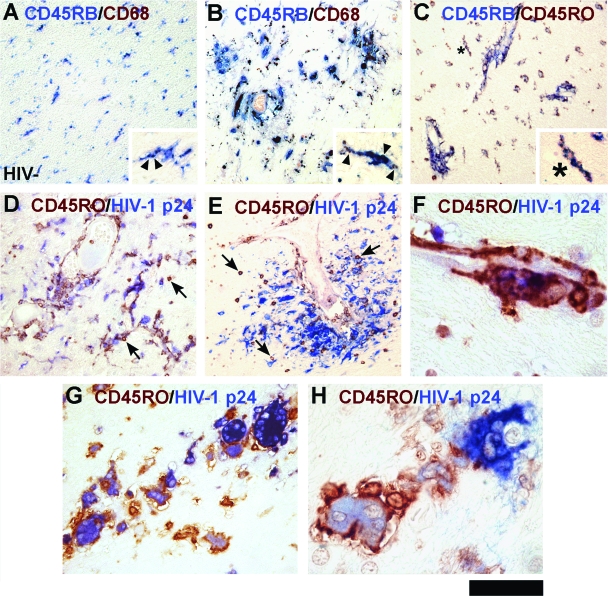
*Analysis of CD45-positive microglia, macrophages and multinucleated giant cells (MGCs).* Double-label studies with macrophage antigens. Note the color of the antibody label matches the color of the chromogen. All panels show HIVE brain except **A** (HIV− control). Microglia in normal brain are double positive for CD45RB/CD68 (**A**). In HIVE, perivascular macrophages and microglia are double positive for CD45RB/CD68 (**B**). CD68 (brown) is intracellular (arrowheads) while CD45 is on the cell surface (insets, **A** and **B**). Microglia double positive for CD45RB and CD45RO in HIVE (**C**, high power view in inset). Intracellular HIV-1 p24 stain marks productively infected cells including MGCs (**D–H**). Low power showing CD45RO+/p24+ microglia in diffuse (**D**) and focal (**E**) distribution. Note that lymphocytes are CD45RO+ but p24−. A high power view of CD45RO+/p24+ perivascular macrophages (**F**). Most p24+ MGCs are strongly CD45RO+ (**G**, **H**). Scale bar represent 300 µm (**A–E**), 30 µm (**F** and **H**), 75 µm (**G**) and 60 µm (insets of **A–C**).

**Figure 3:**
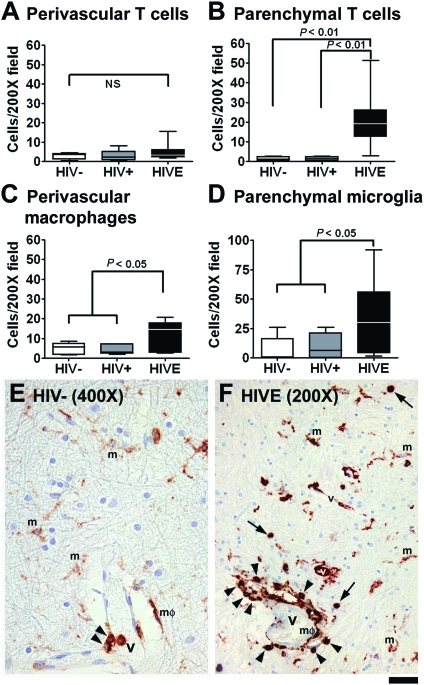
*Quantitative analysis of CD45RO expression.* CD45RO+ cells were counted at 200× based on cell type (**A–D**). Examples of each cell type are shown in **E** (HIV−) and **F** [HIV encephalitis (HIVE)]. Arrows indicate parenchymal lymphocytes; arrowheads, perivascular lymphocytes; m, microglia; and mΦ, macrophages. **A** and **B**. No difference was noted in the RO+ perivascular T cell counts among groups, while the RO+ parenchymal T cell count was significantly elevated in HIVE compared with either control group by ANOVA. **C** and **D**. The number of perivascular macrophages and parenchymal microglia were also significantly elevated in HIVE compared with none-HIVE (combined HIV−/HIV+, *t*-*test*). The scale bar represents 25 µm in **E** and 50 µm in **F**.

### Quantitative analysis of CD45 isoform expression (Figure 2)

The αRA, αRC, αRB and αRO-reactive cell counts in the three patient groups (HIV−, HIV+ and HIVE) are shown in Figure 2. αCD45RA- and αRC-reactive cells were rare averaging—one cell per 400× field in HIVE and even fewer in control brains (Figure 2A,B). Neither RA nor RC counts were significantly different among groups (NS = not significant). As RB+ and RO+ cells included heterogeneous cell populations, they were further divided into parenchymal (Par) and perivascular (PV) populations and the numbers were compared. Because of constitutive expression in normal microglia, CD45RB parenchymal counts were uniformly high in all three groups (*P* > 0.05), averaging ∼50 per 400× field (Figure 2C). By contrast, perivascular RB counts were significantly elevated in HIVE compared with HIV− (*P* < 0.001) or HIV+ (*P* < 0.01). The total RB counts in HIVE, but showed an upward trend, were not significantly elevated. CD45RO counts were also determined in 400× field for comparison. Overall, RO counts were considerably lower than RB counts in all patient groups (Figure 2D). When RO counts were compared between groups, the parenchymal (but not perivascular) RO counts in HIVE were significant elevated compared with the HIV+ group. The total RO counts were also significantly elevated in HIVE compared with HIV+ (also see below).

### Cell type-specific quantitation of CD45RO expression (Figure 3)

As CD45RO expression alone was significantly elevated in HIVE, we further analyzed RO expression on different cell types in HIVE and control brains. RO+ cells were enumerated in 200× microscopic field according to their morphology and location. Examples are shown in Figure 3E,F: (i) perivascular lymphocytes (arrowheads); (ii) parenchymal lymphocytes (arrows); (iii) perivascular macrophages (mΦ); and (iv) parenchymal microglia (m). While microglia are ramified, perivascular macrophages are round or elongated in shape. The identity of these cells is confirmed by double labeling for T cell and macrophage markers (see Figures 5 and 6 below). The results show that the number of CD45RO+ cells increased in HIVE in all categories, except for perivascular lymphocytes (Figure 3A–D). Because significant inflammatory cell (macrophages and lymphocytes) infiltration occurs only in HIVE, the increase in CD45RO+ T cell and macrophage number in HIVE represents the presence of these new cell infiltrates (Figure 3A–C). On the other hand, the increase in microglial CD45RO in HIVE represents that a larger proportion of microglia are positive for CD45RO (Figure 3D). The staining intensity in RO+ microglia appears to have increased in HIVE as well (see Figure 3E,F, for example), but our analysis only reflected the CD45+ cell numbers.

### Relative CD45RB and RO expression on different cell types based on immunohistochemical detection methods with different sensitivity (Figure 4)

**Figure 4:**
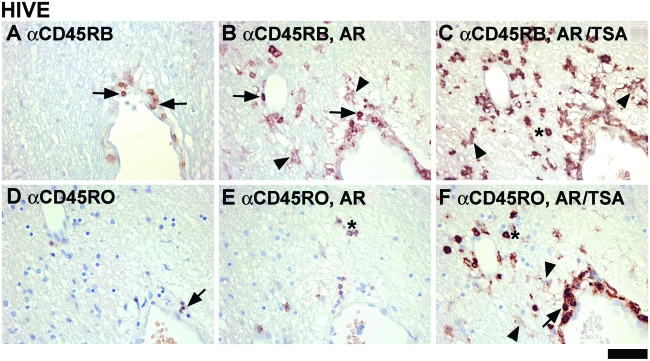
*Comparison of immunohistochemistry (IHC) methods: the effect of antigen retrieval and tyramide signal amplification (TSA) on CD45 detection.* Six serial sections of the same case were immunostained for CD45RB (top panel) and CD45RO (bottom panel) under three different conditions: no treatment (**A** and **D**), antigen retrieval (AR) (**B** and **E**), and AR plus TSA (**C** and **F**). Without AR, CD45RB or CD45RO immunoreactivity is shown in lymphocytes only (arrows, **A** and **D**). With AR, lymphocyte staining for RB (arrows in **B**) and RO (asterisk in **E**) is increased (RB > RO) and some microglial staining becomes detectable (arrowheads in **B**). AR plus TSA further enhances RB staining in microglia (arrowheads in **C**) and lymphocytes (asterisk in **C**) and RO staining for all cells (microglia indicated with arrowheads in **F**). The scale bar represents 50 µm.

Reports of αCD45 labeling of microglia vary (1, 7, 13, 24, 33, 46, 56). We tested immunohistochemical techniques with differing sensitivity to determine whether microglial CD45 immunoreactivity varies depending on the technique and cell type. AR in the form of boiling of the paraffin sections in sodium citrate buffer is commonly used to increase the antigen detection. TSA is another method that increases the sensitivity of staining by up to two logs (21, 47). We have compared three conditions (no treatment, AR alone, and AR plus TSA) for detection of RB and RO. Examples from an HIVE case are shown in Figure 4. Both RB and RO stains are limited to lymphocytes without AR or TSA (Figure 4A,D). With AR, lymphocyte staining is intensified and some non-lymphocyte stain becomes detectable, especially for RB (Figure 4B,E). With AR plus TSA, lymphocyte staining is greatly intensified and more microglia and macrophages become detectable (Figure 4C,F). CD45RB staining intensity tends to be greater than CD45RO staining, particularly on non-lymphoid cells [compare top (RB) and bottom (RO) panel], regardless of the HIV status (not shown).

### CD45RB and CD45RO in macrophages and microglia (Figure 5)

To confirm the identity of CD45+ cells, we performed double labeling with macrophage markers (CD68) or T cell markers (CD3). Figure 5A demonstrates overlapping of CD68+ and CD45RB+ populations in control HIV− brain confirming that they are microglia. Figure 5B demonstrates CD68+/CD45RB+ cells in perivascular and parenchymal locations (HIVE) signifying macrophages and microglia. Double labeling for CD45RO and CD45RB showed that all CD45RO+ microglia were also CD45RB+ (Figure 5C, HIVE). Many of the CD45RO+ cells were also HIV-1 p24+ in HIVE (Figure 5D–H). Exceptions were lymphocytes that were CD45RO+ but p24− (Figure 5D,E: small, round, brown cells throughout). We find most MGCs to be p24+ and CD45RO+ (Figure 5F–H), though RO− MGCs were also observed (Figure 5H, MGC to the right). For instance, cell counting in one case showed 83% of a total of 57 MGCs to be CD45RO+/p24+ and 17% CD45RO−/p24+.

### Most lymphocytes in HIVE are CD8+ (Figure 6)

**Figure 6:**
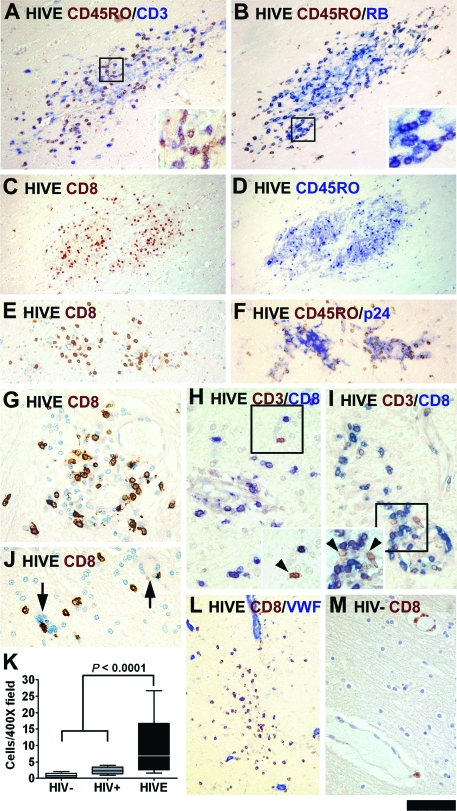
*Characterization of T cell infiltrates in HIV encephalitis (HIVE).* Immunolabeled serial sections of three different microglial nodules are displayed. The chromogen marking the antibody is indicated by the color of the text. The first set shows CD45RO+/CD3+ (**A**) or CD45RO+/RB+ (**B**) small round lymphocytes. Double-labeled cells appear darker than single-labeled cells (see insets). CD8+ cells are observed in microglial nodules, but are also scattered throughout the parenchyma (**C–K**). Serial sections of two microglial nodules indicate that the number and distribution of CD8+ and CD45RO+ cells correspond (**C–F**). CD8+ cells are seen adjacent to p24+ cells (**F**) and unstained MGCs (arrows in **J**). To verify the identity of the T lymphocytes, double labeling for CD3 and CD8 was performed and the majority are CD3/CD8 double labeled (**H**, **I**). CD3+/CD8− cells (brown, arrowheads, insets) are noted but rare. The parenchymal position of most CD8+ cells in HIVE is evident in sections labeled for an endothelial antigen, Von Willebrand Factor (VWF, **L**). CD8+ cells in control brains are limited in the perivascular distribution (**M**). CD8+ cell counts are increased in HIVE compared with combined control groups by *t*-*test*. The scale bar represents 120 µm (**A**, **B**, **E**, **F**, **L**), 240 µm (**C** and **D**), 60 µm (**G–J** and **M**) and 50 µm (all insets).

To ascertain the identity of CD45RO+ and CD45RB+ lymphocytes, we stained the sections for CD3 (pan T-cell) and CD8. Multiple attempts at CD4 staining did not produce reliable results. Thus, we inferred CD4 expression from CD3/CD8 double-labeled sections (CD3+/CD8−). Double labeling showed that CD45RO+ lymphocytes were also CD3+ (Figure 6A) and CD45RB+ (Figure 6B). We compared the number and distribution of CD8+ cells and CD45RO+ lymphocytes in single-stained serial sections. Low and high power views of a microglial nodule (Figure 6C–E) demonstrate that CD8+ and CD45RO+ cell distributions overlap, suggesting that lymphocytes in HIVE are CD8+ T cells. The lymphocytes (CD45RO+, CD8+) were in close contact with p24+ MGCs, but they themselves were p24− (Figure 6F, also see J). Double staining revealed that the majority of CD3+ cells were also CD8+ (Figure 6H,I). Quantitation in four HIVE cases showed that 97.5% ± 1.0% (mean ± SEM) of CD3+ cells were CD8+. CD3+/CD8− cells (presumably CD4+ T cells) were detected, but very rarely (Figure 6H,I). Double labeling with an endothelial specific antigen [(Von Willebrand Factor (VWF)] confirmed the parenchymal location of CD8 cells in HIVE (Figure 6L). In control brains, CD8+ cells were few and limited to the perivascular region (Figure 6M). CD8+ cell counts were determined as average in six 400× fields and showed a significant increase in HIVE over combined control groups (Figure 6K).

## DISCUSSION

In this study, we demonstrate that the predominant CD45 isoforms expressed in HIVE and control brains are CD45RB and CD45RO. Based on the reactivity to exon-specific antibodies we determined that of the four RB-containing isoforms (ABC, AB, BC, B), the αRB-reactive isoform in macrophages and microglia is CD45RB and not ABC, AB or BC. The predominant αRB-reactive isoform in CNS-infiltrating lymphocytes is also RB, since RA- or RC-expressing cells were extremely rare. CD45RO was also detected in all three cell types. The pattern of CD45 isoform expression in microglia in human CNS is similar to that *in vitro*. Cultured human microglia contain abundant CD45RB, and a low level of CD45RO, but no αRA- or αRC-reactive isoforms (25). The robust expression of CD45RB in microglia in normal human brain supports the potential usefulness of CD45RB as a marker of resting microglia, since the majority of widely used markers identify activated cells (19, 36). In addition, CD45’s membrane expression is particularly useful in delineating the relationship between microglia and adjacent neural cell types, such as neurons (see Figure 1J, for example).

Because of the constitutive expression of CD45RB in normal microglia, quantitative analysis did not reveal that CD45RB counts were significantly increased in HIVE. Unlike CD45RB, the CD45RO counts showed a significant increase in HIVE. When CD45RO counts were analyzed according to the cell type, this increase was due to RO+ T cells, macrophages as well as microglia. While the expression of CD45RO in CNS-infiltrating T cells is well known (55), its expression in microglia and macrophages is less well appreciated. We find that highly sensitive TSA amplification was required for detection of microglial and macrophage CD45RO. Unlike CD45RB, CD45RO expression in control microglia (in both HIV− and HIV+ brains) was variable. Although there is a high overlap between HIV-1 p24 expression and CD45RO reactivity in macrophages and microglia, the increase in CD45RO reactivity is unlikely to be unique to HIVE. Indeed, CD45RO+ microglia have been found in Alzheimer’s brains concentrated in senile plaques (1).

Initially created for immunoassays (3), the TSA system utilizes catalyzed reporter deposition technique that enhances immunolabeling by 10- to 100-fold (21, 39). Previously, staining for CD45 antigen has been performed without the TSA method (1, 13, 33, 36, 38, 44). We observe that TSA was invaluable in detecting CD45RO on *both* macrophages and microglia, while detection of CD45RO on T cells did not require TSA. These results suggest that CD45RO expression in T cells must be at least one to two orders of magnitude higher than that in macrophages or microglia. Similar results are found in FACS analysis studies of *ex vivo* cells which showed higher levels of CD45 in lymphocytes than in monocytes or microglia (9). In our analysis of HIVE sections, we find the majority of strongly CD45+ inflammatory cells to be T cells rather than monocytes or macrophages. These results contrast with those of others who found monocytes and macrophages to be the predominant cells with high CD45 expression (13, 18). Some of the strongly CD45+ cells in these studies appear to have been misidentified as macrophages, as they have the typical morphology of lymphocytes. We find it difficult to definitively identify CD45+ monocytes and macrophages in HIVE without simultaneously labeling for T cell markers.

We observe that the number of T cells infiltrating HIVE brain can be very high. Surprisingly, a substantial number of T cells were within the brain parenchyma often associated with HIV-1 infected cells and/or microglial nodules. We also found the majority of T cells in HIVE to be CD8+, in agreement with previous reports (26, 34). Similar findings were reported for simian immunodeficiency virus encephalitis (26, 32) and may in part reflect the general CD4+ T cell deficiency in AIDS. However, CD8+ infiltrates devoid of CD4+ T cells have been reported in immune-restored post-HAART individuals (34). The predominance of CD8+ cells found in this and other studies predicts that T cells are unlikely to be reservoirs of HIV-1 within the CNS (44, 55).

We have demonstrated that, despite the generally low level of microglial CD45RO, triggering this molecule with an antibody (UCHL-1) *in vitro* has a profound effect in human microglia. αCD45RO virtually completely suppressed GM-CSF-induced microglial proliferation (48) and potently suppressed HIV-1 replication (25). Interestingly, αCD45RB antibodies in human microglia did not induce any activity (25, 48), whereas in the mouse, α-CD45RB suppressed microglial activation and induced tolerance to transplantation rejections (49, 50, 53). Given the robust expression of CD45RB in human microglia, these results reflect the effectiveness of individual antibodies rather than species-dependent differences in the expression of CD45 isoforms. The expression of CD45RB in human cells suggests that this CD45 isoform may also be targeted with an effective antibody.

Our study showing CD45 expression in microglia may have clinical applications. CD45 has been shown to be functionally important in murine models of Alzheimer’s disease (49), and microglial CD45 may be a potential therapeutic target in inflammatory CNS diseases, as has been proposed for systemic immunological and neoplastic diseases (23). Natural CD45 ligands have not been identified despite extensive searches (16) but CD45 tyrosine phosphatase activity can be modulated by antibodies, RNAi or gene deletion (22, 23, 40, 48). Inhibition of CD45 expression by the latter two methods have resulted in an activated cell phenotype, indicating that CD45 normally functions to maintain the suppressed cell phenotype (22, 49). Therefore, an antibody approach remains an ideal option to target CD45 to suppress cell activity and inflammation. Our work suggests a therapeutic potential for αCD45RO for AIDS dementia and possibly for other inflammatory and neurodegenerative disorders in which suppression of microglial activation is desirable.

## Abbreviations:

LCA: leukocyte common antigen
VWF: VWF = Von Willebrand Factor.
